# A spectral CT-derived metrics-based radiomics model in differentiating *de novo* osteoblastic bone metastasis and bone islands in newly diagnosed cancer patients

**DOI:** 10.3389/fonc.2025.1535860

**Published:** 2026-01-13

**Authors:** Qian Yang, Honghong Luo, Liyan Zou, Cuiyun Yuan, Kun Ma, Chenbin Liu, Dehong Luo, Zhou Liu

**Affiliations:** 1 Department of Radiology, National Cancer Center/National Clinical Research Center for Cancer/Cancer Hospital and Shenzhen Hospital, Chinese Academy of Medical Sciences and Peking Union Medical College, Shenzhen, China; 2 Department of Radiation Oncology, National Cancer Center/National Clinical Research Center for Cancer/Cancer Hospital and Shenzhen Hospital, Chinese Academy of Medical Sciences and Peking Union Medical College, Shenzhen, China; 3 Computed Tomography (CT) Imaging Research Center, GE Healthcare, Beijing, China

**Keywords:** dual-energy, computed tomography, osteoblastic, bone metastasis, bone island

## Abstract

**Purpose:**

To investigate the value of radiomics features extracted from plain and enhanced spectral CT-derived metrics in differentiating osteoblastic bone metastasis (OBM) and bone island (BI) in newly diagnosed cancer patients.

**Methods:**

From January to November 2020, 51 newly diagnosed cancer patients with 204 bone lesions (OBM = 116, BI = 88) receiving spectral CT were retrospectively enrolled. 40–140 keV mono-energy images were generated from plain CT and contrast-enhanced CT, and material-decomposition images, including water (calcium) and calcium (water) substrate density images from plain CT and Iodine (calcium) substrate density images from contrast-enhanced CT. Radiomics features were extracted from the manually segmented lesions, including shape feature set, material-separation feature set, plain spectral CT feature set, and enhanced spectral CT feature set. U-test and LASSO analysis were sequentially used to select the most relevant features. The shape model, material-separation model, plain CT model, contrast-enhanced CT model, and combined model were built using Random Forest with model performance evaluated using ROC analysis and compared using the Delong test.

**Results:**

After feature selection, four features were selected for the shape set, seven features for the material-separation set, seven features for the plain spectral CT set, and nine features for the enhanced spectral CT set. The AUC of the shape model was significantly smaller than that of the other four models (all *P* < 0.05). The combined model (AUC = 0.874, 95%CI: 0.821-0.916) outperformed the material-separation model (AUC = 0.828, 95%CI: 0.769-0.877, *P* = 0.005), the plain spectral CT model (AUC = 0.820 95%CI: 0.760-0.870, *P* = 0.005) and the enhanced spectral CT model (AUC = 0.838, 95%CI: 0.780-0.886, *P* = 0.005).

**Conclusion:**

The radiomics features derived from spectral CT metrics will enhance the differentiation of *de novo* OBM and BI in newly diagnosed cancer patients.

## Introduction

Incidental hyper-density bone lesions are commonly observed during thoraco-abdominal CT scans, with bone island (BI) occurring in approximately 3.2% of the general population (1) and *de novo* osteoblastic metastasis (OBM) found in around 5.1% of the overall cancer patients population (2). A BI is a focal area of dense bone tissue within the cortical bone, composed of mature lamellar bone, and represents a normal variant of bone tissue (1). In contrast, OBM refers to a pathological condition in which certain malignant tumors spread to the bones via hematogenous dissemination, stimulating osteoblast activity and resulting in localized bone hyperplasia (2). While OBM elevates the clinical stage to stage IV cancer, BI does not need intervention for its benignity. Consequently, differentiating these two entities in newly diagnosed cancer patients is essential in clinical scenarios, as accurate clinical staging based on differential diagnosis would profoundly impact treatment decisions and patient prognoses.

Although both lesions exhibit distinct imaging findings at larger sizes, they could mimic each other at initial diagnosis, appearing as small hyper-dense focal lesions with clear margins. Moreover, the co-existence of BI and OBM can complicate the clinical evaluation of newly diagnosed cancer patients in clinical practice (1). Conventional subjective assessments based on CT imaging findings often suffer from inter-observer variability. Thus, there is a pressing need for a quantitative approach to accurately differentiating BI and OBM in their early stage in the clinical setting.

Initially, several studies have explored the use of CT attenuation to distinguish between BI and OBM, revealing that BI typically exhibits significantly higher CT attenuation values than OBM (3–5). However, the overlap in CT attenuation value between these two entities necessitates additional information for differential diagnosis. Unlike conventional single-energy CT, dual-energy spectral CT offers an innovative approach by utilizing two energy levels to assess materials with different attenuation properties. This technique enables not only the reconstruction of anatomical images but also the generation of quantitative metric maps, including monochromatic spectral images ranging from 40 to 140 keV, material decomposition images, etc. (6). Previously, both Dong et al. and our study have preliminarily demonstrated the potential of a variety of quantitative features derived from spectral CT for differentiating OBM from BI in newly diagnosed cancer patients (7, 8). Nevertheless, the spatial heterogeneity of these quantitative metric maps has yet to be thoroughly investigated.

Previously, Hong et al. developed a radiomics model using conventional CT to differentiate between BI and OBM and validated its robustness (9). To our knowledge, monochromatic spectral images and material decomposition images have not yet been utilized to extract radiomic features to differentiate *de novo* OBM from BI in newly diagnosed cancer patients. Herein, we report on applying the radiomics-based machine learning approach to identify specific radiomics patterns on the dual-energy spectral CT-derived monochromatic spectral images and material-decomposition images induced by OBM and BI and leveraged these distinct radiomic features to differentiate *de novo* OBM from BI in newly diagnosed cancer patients.

## Materials and methods

### Patient enrollment

**Figure 1:**
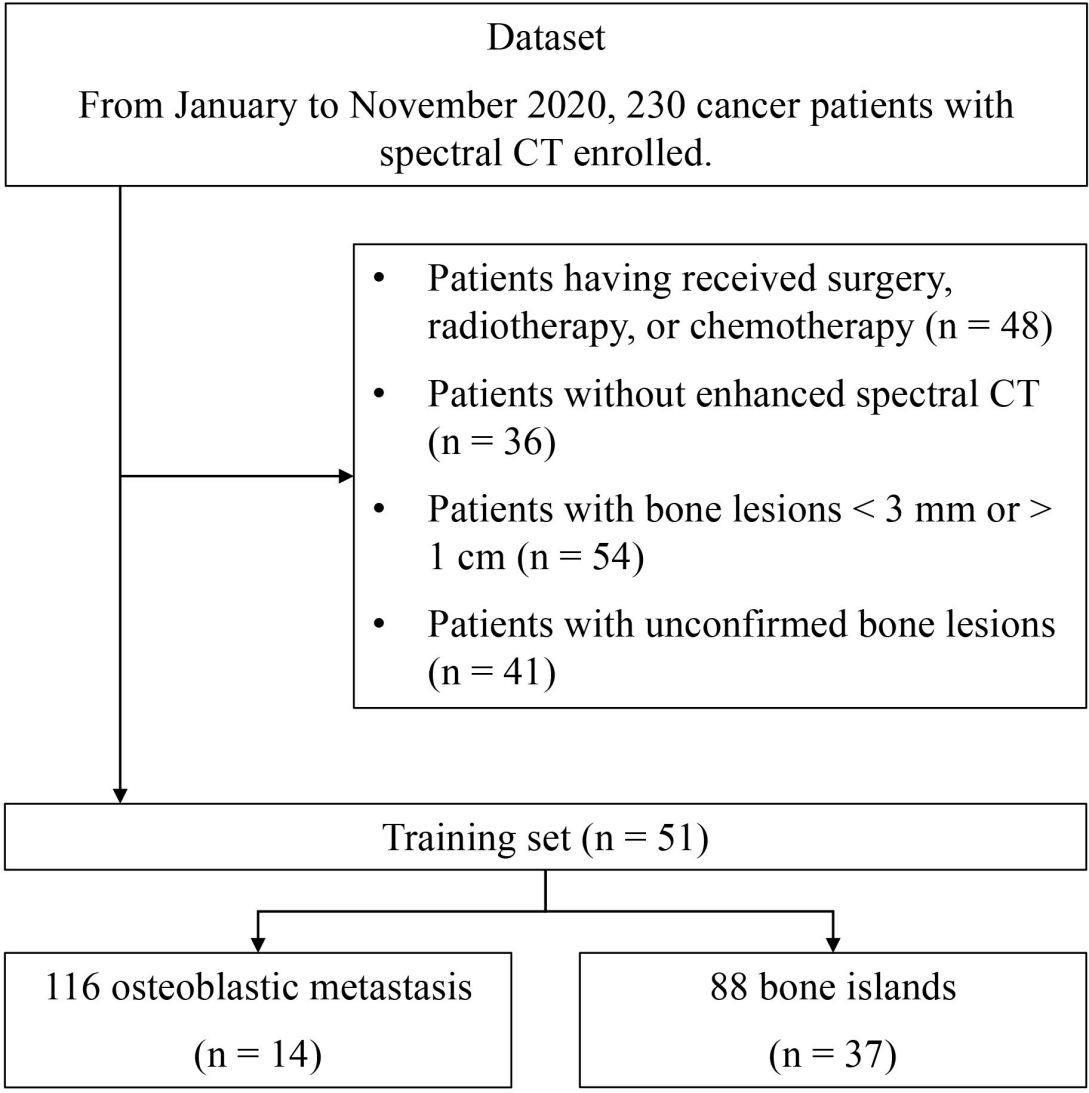
The patient selection process.

This retrospective study received approval from the Institutional Ethics Committee, and informed consent from individual subjects was waived. Between January and November 2020, 230 newly diagnosed cancer patients who underwent dual-energy spectral CT were enrolled. The inclusion criteria included: 1) satisfactory image quality without non-negligible artifacts; 2) hyperdense lesions measuring from 3–20 mm; 3) confirmed diagnosis of either BI or OBM; 4) no prior treatment before the spectral CT scan. Those patients were excluded if: 1) primary cancer cannot be determined; 2) the diagnosis of BI or OBM cannot be determined; 3) lesions were larger than 20 mm or smaller than 3 mm; 4) both osteolytic and osteoblastic metastasis were present on CT; 5) there was evidence of compression fracture or pathologic fracture. After applying these criteria, 51 newly diagnosed cancer patients with 204 bone lesions were ultimately selected for the study. The flowchart of patient selection is depicted in **Figure 1**, and the clinical characteristics of enrolled patients are detailed in **Table 1**.

**Table 1:** Clinical characteristics of selected patients.

Dataset	BI (n = 37)	OBM (n = 14)	*P* value
Gender (male: female)	23: 14	1: 1	0.391
Age (year)	59.97 ± 12.61	57.71 ± 10.97	0.579
Size (mm)			
Short axial diameter	3.99 ± 1.37	7.99 ± 4.40	< 0.001*
Long axial diameter	5.75 ± 2.07	9.91 ± 4.84	< 0.001*
Lesion location	N = 88	N = 116	
Vertebrae	25	73	
Scapula	2	4	
Rib	12	5	
Extremities	9	3	
Sternum	13	1	
Bony pelvis	24	27	
Frontal bone	3	3	
Primary lesion	Lung cancer (19)	Lung cancer (10)	
	Nasopharyngeal carcinoma (3)	Breast carcinoma (2)	
	Colorectal cancer (3)	Vulvar cancer (1)	
	Cervical carcinoma (2)	prostatic cancer (1)	
	Breast carcinoma (1)		
	Stomach cancer (3)		
	Ovarian cancer (1)		
	Esophagus carcinoma (3)		
	Renal carcinoma (1)		
	Lymphoma (1)		

*indicates a statistically significant difference.

### Reference standards

In differentiating BI from OBM, we initially assess lesions using MRI, PET-CT, and bone scintigraphy.

1. For lesions preliminarily diagnosed as bone islands: A 12-month follow-up is conducted.

1) If the lesion remains stable, it is confirmed as a bone island. 2) If interval growth is observed, it is reclassified as an osteoblastic metastasis.

2. For lesions initially suspected to be osteoblastic metastases:

1. If the lesion shows progression during follow-up, the diagnosis of metastasis is confirmed.
2. If no change is observed after 12 months, the lesion is excluded from the study.

### CT imaging acquisition protocol

The research employed a 256-row multidetector CT scanner (Revolution CT 750 HD, GE Healthcare, Waukesha, USA) featuring gemstone spectral imaging (GSI) mode for all examinations. The scanning parameters encompassed a field of view ranging from 40 to 50 cm customized to each patient’s size; a matrix resolution of 512 × 512; voltages of 140 kVp and 80 kVp utilizing an ultra-fast peak kVp switching with 0.5 ms, automated tube current, a helical pitch of 0.992, collimation of 64 ×0.625 mm, rotation time of 0.5 ms, and employing a standard reconstruction kernel. A bolus of contrast agent (Loversol, 350 mg/ml; Jiangsu Hengrui, China) was administered at a standard dosage of 1.5 ml/kg, with a flow rate of 2.0–3.0 ml/s. The venous phase of the contrast-enhanced CT scans was captured 50 seconds after injection. Standard algorithm reconstruction was employed for CT images with a slice thickness of 1.25 mm and an ASIR-V of 50%. The resultant CT images were analyzed by experienced board-certified radiologists (L.Y.Z. and Z.L.), with those unsatisfactory images with a low signal-to-noise ratio or overt motion artifacts excluded.

### Lesion segmentation

On the standard DECT workstation (GSI Viewer, AW4.7,GE Healthcare), raw data was reconstructed into 40–140 keV mono-energy images from non-contrast CT and contrast-enhanced CT, and material-decomposition images using two-material decomposition algorithms, including water (calcium) and calcium (water) substrate density images from non-contrast spectral CT and Iodine (calcium) substrate density images from contrast-enhanced spectral CT. Using ITK-SNAP software (version 3.8.0, http://www.itksnap.org), all of the lesions were manually segmented on consecutive non-contrast spectral CT and contrast spectral CT images slice by slice by a radiologist (H.H.L. with 4 years of experience in bone CT imaging), and then reviewed by a senior radiologist (Z.L. with more than 10 years of experience in bone CT imaging). Any discrepancies were dissolved through discussion.

### Feature extraction

Based on the segmented 3D lesions, radiomics features were extracted using “Pyradiomics” packages for each type of generated image. For each segmented lesion, 14 shape features, 18 histogram features, and 75 texture features were extracted from each of plain single-energy images (40–140 keV), each of enhanced single-energy images (40–140 keV), and each of material-separation images including water (calcium) image, calcium (water) image, and iodine (calcium) image, respectively. Since the shape features extracted were all the same for each type of image, we singled out these features to form an independent shape feature set. Finally, four feature sets were formed, including the shape feature set (n = 14), material-separation feature set (n = [18 + 75] × 3 = 279), plain spectral CT feature set (n = [18 + 75] × 11 = 1023), and enhanced spectral CT feature set (n = [18 + 75] × 11 = 1023).

### Feature selection

U-test (*P* < 0.05) and LASSO analysis were sequentially used to select the most relevant features to the differentiation for each feature set, with the lasso coefficient being used for feature importance ranking.

### Model building

Random forest was used as the classifier to train the machine learning model. In total, we built five models, including a shape model based on the selected shape feature set, a material-separation model based on the selected material-separation feature set, a plain spectral CT model based on the selected plain spectral CT feature set, an enhanced spectral CT model based on the selected enhanced spectral CT feature set, and a combined model based on the combined feature set. To evaluate the classification performance, we tested the model using cross-validation (10-fold) with sensitivity, specificity, accuracy, and area under the receiver operating characteristic curve (AUC) collected.

### Statistical analysis

All continuous variables were denoted as mean ± standard deviation. On SPSS 26.0 (SPSS Incorporation, Chicago, IL), an independent sample student-t test was used to test the difference in demographic data, including age between the BI group and the OBM group. DeLong test (10) was used to compare the performance between each two of the shape model, material-separation model, plain spectral CT model, enhanced spectral CT model, and combined model using MedCalc (version 19.0.4; MedCalc Software). Decision curve analysis (DCA) was applied to quantify the model’s net clinical benefit using the R statistical package (MathSoft Inc., Seattle, WA). Two-tailed *P* < 0.05 indicates a statistically significant difference.

## Results

### Patient characteristics

This study included 51 newly diagnosed cancer patients with 204 bone lesions (116 OBM and 88 BIs). The most common location is the vertebrae both for the BI group (25/88) and the OBM group (73/116). For primary cancer, OBM were predominantly originating from lung cancer (10/14) in our study. There were no significant differences in age and gender between the two groups. However, lesions in the OBM group were significantly larger than those in the BI group (*P* < 0.001). The patient characteristics are shown in **Table 1**.

### Feature selection and ranking

**Figure 2:**
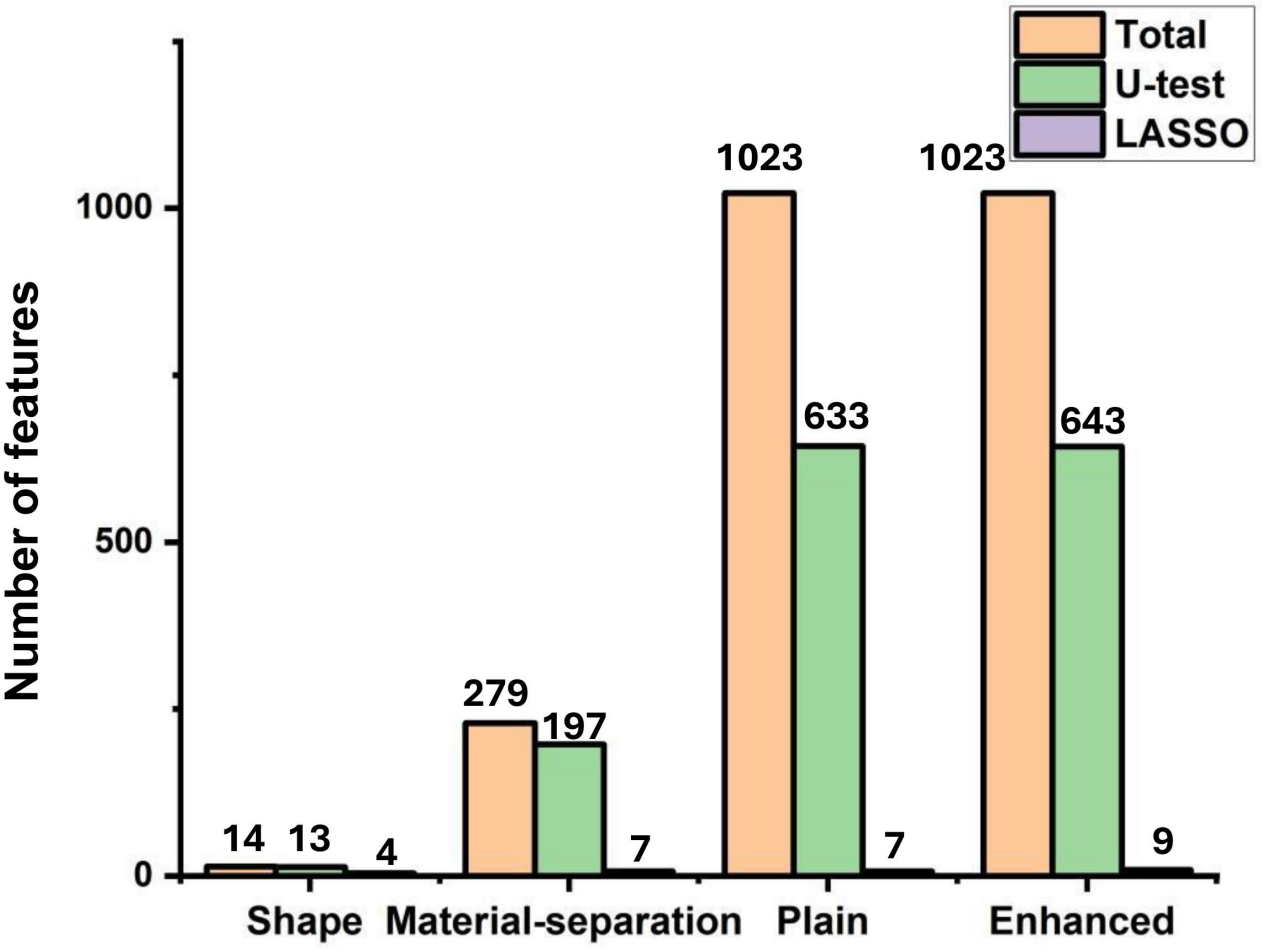
Feature selection process.

Of the 14 shape features, 13 features were selected after U-test and 4 features were selected after LASSO analysis (**Figure 2**). For the material-separation feature set with a total of 279 features, 197 features were selected after U-test, and 7 features were selected after LASSO analysis, including 5 features from calcium (water) image and 2 features from iodine (calcium) image (**Figure 2**).

**Figure 3:**
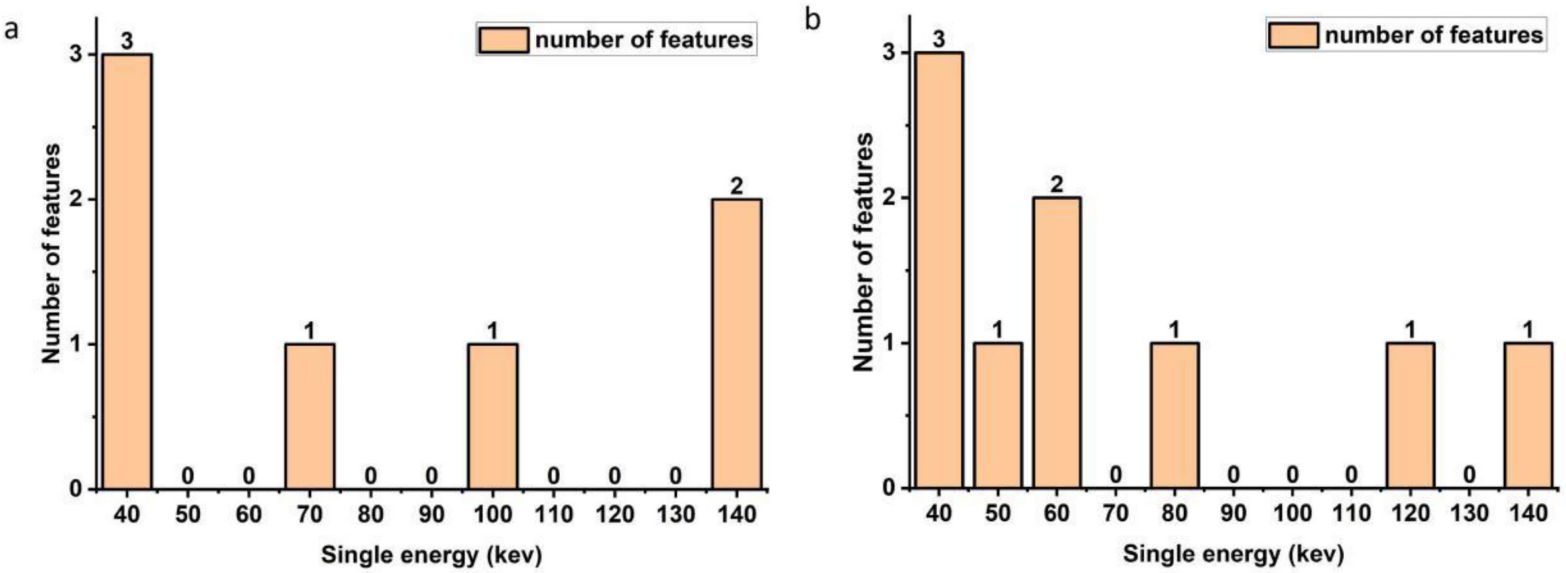
Distribution of features selected from plain spectral CT feature set (a) and enhanced spectral CT feature set.

**Figure 4:**
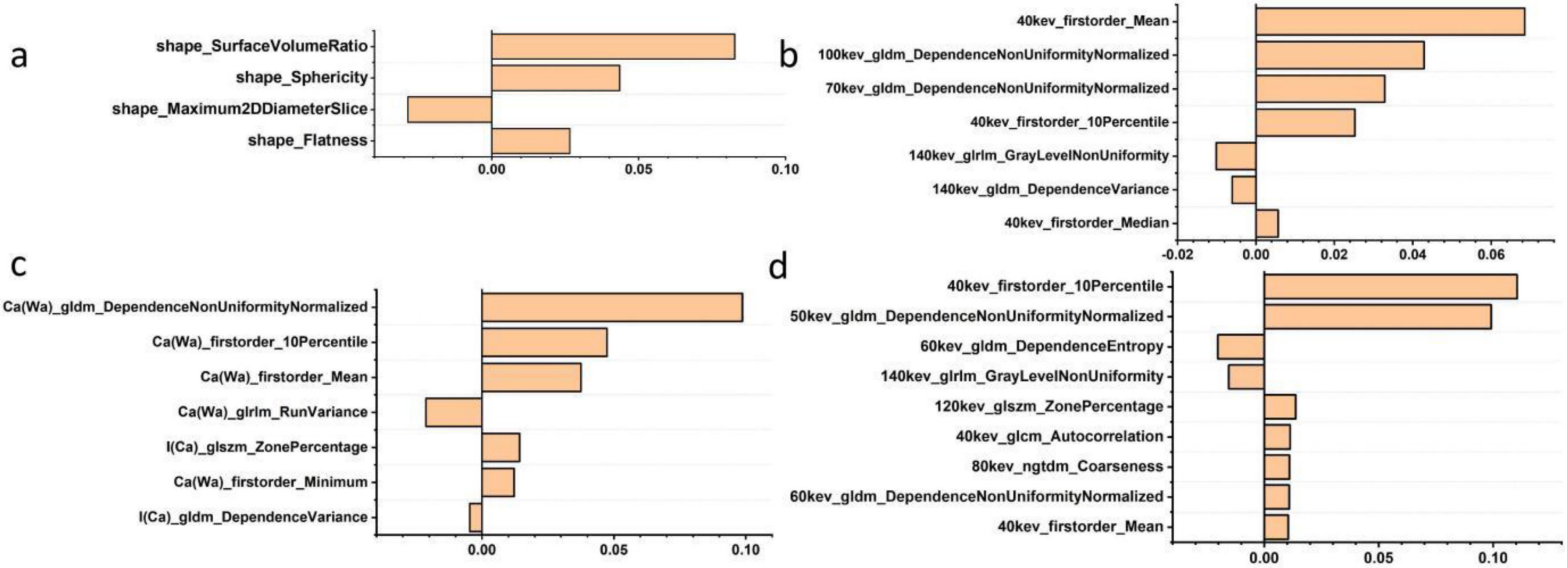
Feature importance ranking based on LASSO coefficient in selected shape feature set **(a)**, plain spectral CT feature set, plain spectral CT feature set **(b)**, material-seperation feature set **(c)**, and enhanced spectral CT feature set **(d)**.

A total of 1023 features were extracted from the plain spectral CT. 644 features were selected after U-test, and 7 features were selected after LASSO analysis in the plain spectral CT feature set (**Figure 2**). These selected features included 3 features extracted from 40 keV CT image, 1 feature from 70 keV CT image, 1 feature from 100 keV CT image, and 2 features from 140 keV CT image. The selected plain single-energy CT features showed a trend of having more features from low and high single-energy images (3 features from 40 keV CT image and 2 features from 140 keV CT image) and fewer from intermediate single-energy images (1 feature from 70 keV and 1 feature of 100 keV (**Figures 3**, **4**).

For the 1023 features in the enhanced feature set, 643 features were selected after U-test, and 9 features were selected after LASSO analysis (**Figure 2**). These 9 features included 3 features from 40 keV CT image, 1 features from 50 keV CT image, 2 features from 60 keV CT image, 1 feature from 80 keV CT image, 1 feature from 120 keV CT image, and 1 feature from 140 keV CT image. However, the selected enhanced single-energy CT features exhibited a trend of having more low-energy features (3 features from 40 keV CT image) and fewer intermediate to high-energy images (1 features from 50 keV CT image, 2 features from 60 keV CT image, 1 feature from 80 keV CT image, 1 feature from 120 keV CT image, and 1 feature from 140 keV CT image) (**Figures 3**, **4**).

In summary, five feature sets were formed, including the shape spectral CT set (n = 4), material-separation spectral CT set (n = 7), plain spectral CT set (n = 7), enhanced spectral CT set (n = 9), and combined feature set (n = 27) (**Figure 4**, **Table 2**).

**Table 2:** Comparison in the selected features between OBM and BI groups.

Features	OBM	BI	P-value
shape_Flatness	-0.168(-2.338~2.293)	0.267(-2.053~2.785)	0.014
shape_Maximum2DDiameterSlice	-0.066(-1.159~4.032)	-0.544(-1.357~3.978)	< 0.001
shape_Sphericity	-0.210(-2.690~1.371)	0.501(-3.923~1.702)	< 0.001
shape_SurfaceVolumeRatio	-0.279(-2.127~2.037)	0.223(-1.781~3.788)	< 0.001
Ca(Wa)_firstorder_10Percentile	-0.615(-1.132~1.820)	0.595(-0.967~3.831)	< 0.001
Ca(Wa)_firstorder_Mean	-0.653(-1.090~1.105)	0.596(-1.092~3.023)	< 0.001
Ca(Wa)_firstorder_Minimum	-0.611(-1.582~1.781)	0.324(-1.132~4.906)	< 0.001
Ca(Wa)_gldm_DependenceNonUniformityNormalized	-0.599(-1.015~1.559)	0.532(-0.862~3.734)	< 0.001
Ca(Wa)_glrlm_RunVariance	-0.008(-0.825~4.927)	-0.730(-0.907~0.997)	< 0.001
I(Ca)_gldm_DependenceVariance	0.354(-1.095~2.863)	-0.827(-1.093~1.450)	< 0.001
I(Ca)_glszm_ZonePercentage	-0.659(-1.101~3.228)	0.537(-1.026~2.498)	< 0.001
40kev_firstorder_10Percentile	-0.339(-2.269~1.638)	0.627(-2.269~3.266)	< 0.001
40kev_firstorder_Mean	-0.607(-1.840~1.109)	0.678(-2.024~2.883)	< 0.001
40kev_firstorder_Median	-0.563(-1.830~1.467)	0.622(-1.890~3.085)	< 0.001
70kev_gldm_DependenceNonUniformityNormalized	-0.657(-1.133~1.520)	0.446(-1.028~2.848)	< 0.001
100kev_gldm_DependenceNonUniformityNormalized	-0.675(-1.076~2.977)	0.545(-0.994~3.331)	< 0.001
140kev_gldm_DependenceVariance	0.188(-1.148~3.427)	-0.835(-1.151~2.006)	< 0.001
140kev_glrlm_GrayLevelNonUniformity	-0.062(-0.680~7.564)	-0.530(-0.674~0.952)	< 0.001
40kev_firstorder_10Percentile	-0.466(-1.988~1.063)	0.722(-1.901~3.135)	< 0.001
40kev_firstorder_Mean	-0.660(-1.855~0.919)	0.838(-1.888~2.607)	< 0.001
40kev_glcm_Autocorrelation	-0.585(-0.747~1.843)	0.021(-0.751~4.543)	< 0.001
50kev_gldm_DependenceNonUniformityNormalized	-0.715(-1.253~1.380)	0.781(-1.150~2.933)	< 0.001
60kev_gldm_DependenceEntropy	0.160(-2.249~2.785)	-0.185(-3.864~2.140)	0.016
60kev_gldm_DependenceNonUniformityNormalized	-0.694(-1.207~1.332)	0.734(-1.196~2.848)	<0.001
80kev_ngtdm_Coarseness	-0.416(-1.015~2.816)	0.108(-0.973~7.986)	<0.001
120kev_glszm_ZonePercentage	-0.654(-1.258~2.020)	0.848(-1.175~2.220)	<0.001
140kev_glrlm_GrayLevelNonUniformity	-0.513(-0.614~0.894)	-0.089(-0.629~7.572)	<0.001

### Model performance

**Figure 5:**
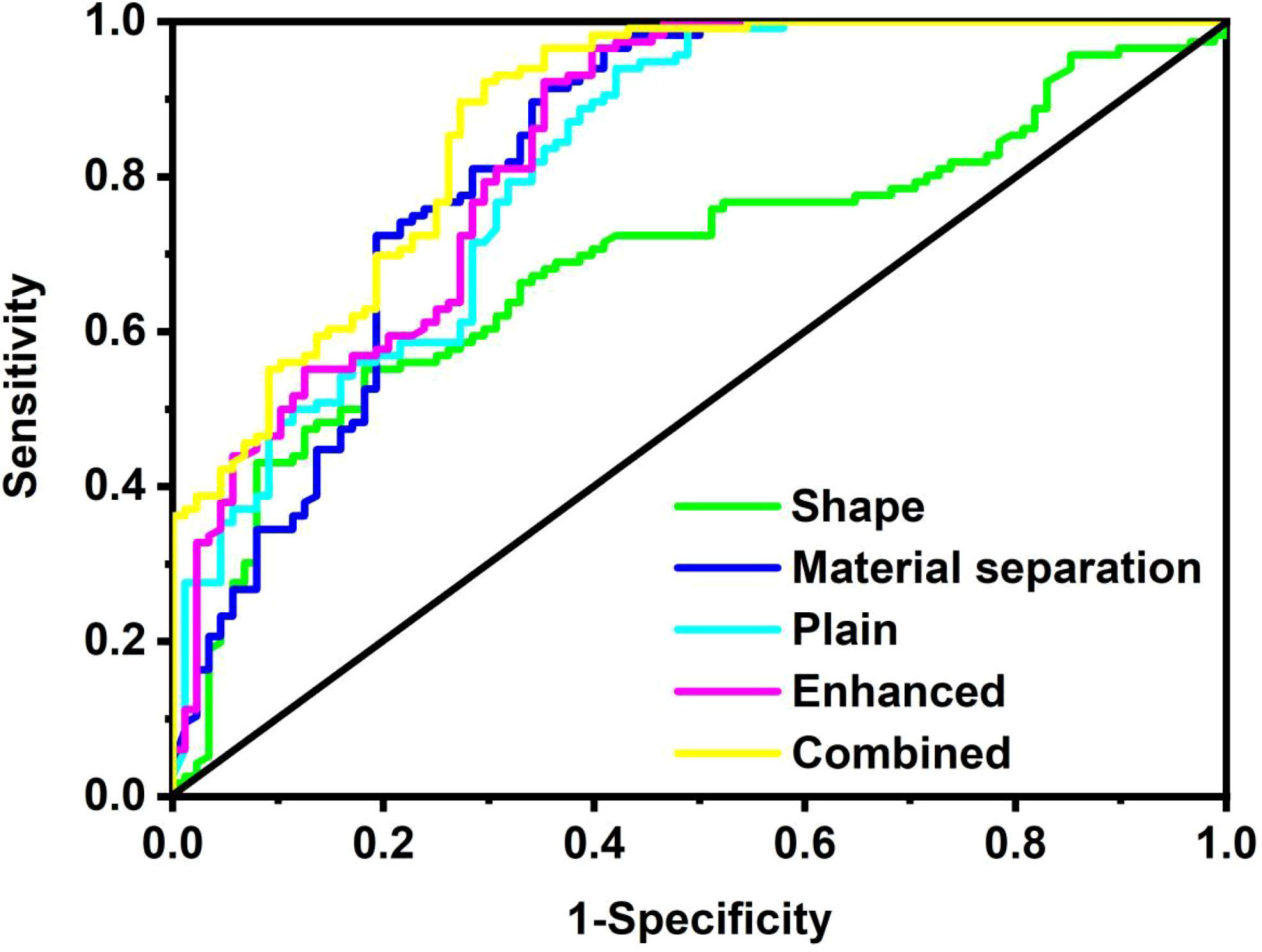
Performance of various spectral CT metrics-based radiomic models.

**Figure 6:**
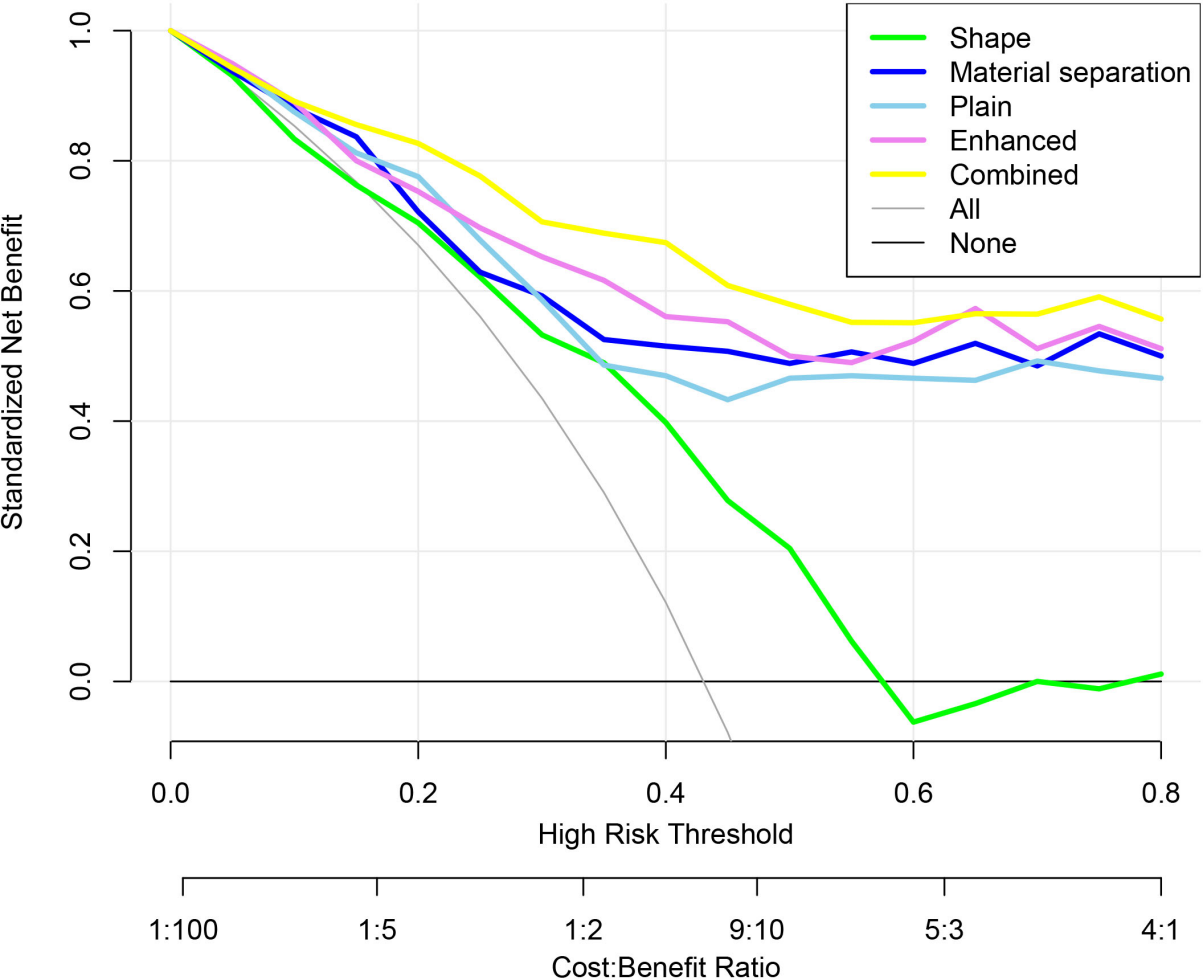
DCA of various spectral CT metrics-based radiomic models.

On the cross-validation, the AUC of the shape model (AUC = 0.690, 95%CI: 0.622-0.753) was significantly smaller than that of the other four models (all *P* < 0.05). The combined model (AUC = 0.874, 95%CI: 0.821-0.916) outperformed the material-separation model (AUC = 0.828, 95%CI: 0.769-0.877, p = 0.005), the plain spectral CT model (AUC = 0.820 95%CI: 0.760-0.870, *P* = 0.005) and the enhanced spectral CT model (AUC = 0.838, 95%CI: 0.780-0.886, *P* = 0.005). There was no significant difference in performance between the material-separation model and the plain spectral CT model (*P* = 0.567), and between the material-separation model and the enhanced spectral CT model (*P* = 0.637), and the plain spectral CT model and the enhanced spectral CT model (*P* = 0.465) (**Figure 5**; **Tables 3**, **4**). Within the risk threshold range of 0-0.8, the net benefit remained positive, demonstrating clinical utility. The combined model showed superior net benefit compared to other models across this threshold spectrum (**Figure 6**).

**Table 3:** Performance of various spectral CT metrics-based radiomic models.

Cross validation	AUC (95% CI)	Sensitivity	Specificity	Accuracy
Shape model	0.690 (0.622-0.753)	0.659	0.664	0.662
Material-separation model	0.828 (0.769-0.877)	0.625	0.922	0.794
Plain spectral CT model	0.820 (0.760-0.870)	0.580	0.922	0.775
Enhanced spectral CT model	0.838 (0.780-0.886)	0.602	0.957	0.804
Combined model	0.874 (0.821-0.916)	0.648	0.948	0.819

**Table 4:** Model performance comparison between each two of those models.

Cross validation	*P*-value
Shape model *vs*. Material-separation model	0.003*
Shape model *vs*. Plain spectral CT model	0.005*
Shape model *vs*. Enhanced spectral CT model	0.001*
Shape model *vs*. Combined model	< 0.001*
Material-separation model *vs*. Plain spectral CT model	0.567
Material-separation model *vs*. Enhanced spectral CT model	0.637
Material-separation model *vs*. combined model	0.005*
Plain spectral CT model *vs*. Enhanced spectral CT model	0.465
Plain spectral CT model *vs*. combined model	0.005*
Enhanced spectral CT model *vs*. combined model	0.005*

*indicates a statistically significant difference.

## Discussion

In the clinical setting, it is challenging to distinguish *de novo* OBM and BI in newly diagnosed cancer patients, especially when the size of the lesion on CT is small. In this study, we developed several machine learning models based on radiomics features extracted from spectral CT image-derived metrics to differentiate *de novo* BI and OBM in newly diagnosed cancer patients. We found that the combined model significantly outperformed the shape model, material-separation model, plain spectral CT model, and enhanced spectral CT model alone, suggesting features extracted from different spectral CT image-derived metrics complement each other, and all contribute to the differentiation.

In histology, unlike BI characterized by mature compact bone distributed in cancellous bone (spongiosa) (11), OBM comprises many tumor cells residing in osteoid tissue with calcium salts deposition (12). Correspondingly, a number of studies using conventional CT have demonstrated that BI had significantly higher mean CT values than that of OBM (3–5). Additionally, both the study by Dong et al. and our previous study using dual-energy spectral CT had confirmed that compared with OBM, BI had significantly higher mean CT values at single energy but with considerably larger standard deviation, suggesting the significant overlap in CT values between these two entities (7, 8). However, the mean CT value at 40 keV was finally selected as one of the most relevant features among 1203 features both for plain and enhanced spectral CT feature sets, suggesting that CT value remains one of the most important features to differentiate BI and OBM despite its overlap. Besides, interestingly, it seems more features were selected from 40 keV images, possibly due to its higher contras (13), which needs further confirmation in future studies.

Typical findings suggesting BI include a sharp margin with thorny, radiating bony spicules on CT (14, 15). However, small BI often mimics OBM at initial diagnosis, especially for newly diagnosed cancer patients, because both could appear as small focal hyper-dense lesions with well-defined margins, which frequently poses a dilemma in clinical practice. Previously, the study by Hong et al. showed that shape-related features-based model had a suboptimal performance with an AUC of only 0.51 (9). Consistently, only four features were finally selected in the shape feature set, and the shape model showed significantly inferior performance compared to models based on plain and enhanced monoenergetic images and material-separation images. For shape features, BI demonstrated significantly higher values than OBM in flatness, sphericity, and surface-volume ratio (all *P* < 0.05). This morphological regularity likely reflects homogeneous composition and well-circumscribed growth pattern for BI. However, both Hong et al. and our study enrolled lesions within 2 cm, suggesting the limited role of shape features in differentiating small BI and OBM.

With its well-defined algorithms, radiomics could take advantage of the spatial heterogeneity of each voxel within the lesions and thus characterize disease-induced image abnormalities and the underlying pathophysiology in much greater details with a high-throughput features (16). Previously, Hong et al. preliminarily demonstrated the usefulness of radiomics features in discriminating BI and OBM, which showed considerably superior performance compared to CT attenuation-based features and shape features. Consistently, in our study, markedly more texture features were selected finally in the plain spectral feature set and enhanced spectral feature set, suggesting the dominant contribution of texture features to the differentiation diagnosis. This likely reflects OBM’s complex microstructure from osteoclast-driven tumor growth versus BI’ homogeneous mature bone architecture. The different texture patterns induced by BI and OBM could be used to improve the accuracy of differential diagnosis further substantially.

Last but not least, based on the histology, we speculate that BI have significantly higher calcium concentrations compared to OBM. Consistently, both Dong et al. and our previous study showed that BI had significantly higher calcium (water) density than that of OBM. In line with the previous studies, 5 out of the 7 finally selected features were extracted from calcium (water) density image, indicating the considerably higher contribution from spatial heterogeneity of calcium distribution than that of iodine distribution after enhancement to the differential diagnosis. For material-separation features, first-order features (mean, minimum, 10th percentile) were consistently elevated and texture analysis revealed markedly higher dependence non-uniformity normalized values in BI for Calcium(Water). These findings correlate with bone islands’ histopathological architecture - mature cortical bone growing along trabeculae with homogeneous calcium distribution. With its unique advantage, dual-energy material-separation CT images could map the spatial heterogeneity of calcium distribution that could be quantified using radiomics features, making the combination of material-separation CT images and radiomics a powerful tool that could be translated into various clinical applications.

This study has the following limitations. Firstly, it is a retrospective study conducted in a single study. Moreover, the sample size is relatively small, and thus external independent testing was not performed in this study. Secondly, we only select lesions with diameters ranging from 3 mm to 2 cm, which might lead to selection bias. However, we chose the lesions in this size range because both types of lesions within this size range usually mimic each other with similar image appearance on CT.

## Conclusion

The radiomics features derived from spectral CT metrics will enhance the differentiation of *de novo* OBM and BI in newly diagnosed cancer patients.

## Data Availability

The raw data supporting the conclusions of this article will be made available by the authors, without undue reservation.
